# Sleep-Disordered Breathing and Clinical Presentation in Infants with Congenital Laryngomalacia: A Polysomnographic Study

**DOI:** 10.3390/jcm14196844

**Published:** 2025-09-27

**Authors:** Sergii Bredun, Anatoli L. Kosakovskiy, Krzysztof Trzpis, Jakub Sroczyński, Anna Wiśniewska, Beniamin Oskar Grabarek, Piotr Żychowski, Jarosław Szydłowski

**Affiliations:** 1 Department of Pediatric Otolaryngology, Poznan University of Medical Sciences, 60-572 Poznan, Poland; ktrzpis@ump.edu.pl (K.T.); jsroczynski@ump.edu.pl (J.S.); pzychowski@op.pl (P.Ż.); jszydlow@ump.edu.pl (J.S.); 2 Doctoral School, Poznan University of Medical Sciences, 60-572 Poznan, Poland; 3 Department of Otorhinolaryngology, Pediatric Otorhinolaryngology and Surdology, Shupyk National Healthcare University of Ukraine, 04112 Kyiv, Ukraine; alkoss@ukr.net; 4 Department of Anatomy, Poznan University of Medical Sciences, 60-572 Poznan, Poland; awisniewska@ump.edu.pl; 5 Collegium Medicum, WSB University, 41-300 Dąbrowa Górnicza, Poland; bgarabek7@gmail.com; 6 Faculty of Medicine and Health Sciences, Andrzej Frycz Modrzewski University in Cracow, 30-705 Cracow, Poland

**Keywords:** laryngomalacia, polysomnography, obstructive sleep apnea, stridor, infant surgery, apnea–hypopnea index, feeding difficulty, congenital airway disorder

## Abstract

**Background/Objectives:** Congenital laryngomalacia (LM) is the most common cause of stridor in infants, presenting with a clinical spectrum that ranges from benign, self-limiting symptoms to severe airway obstruction. This study aimed to objectively characterize the type and severity of sleep-disordered breathing in infants with LM using polysomnography (PSG) and to correlate findings with LM subtypes, clinical presentation, and type of surgical intervention. **Methods:** A cohort of 42 infants diagnosed with LM (Type I: *n* = 14, Type II: *n* = 18, Type III: *n* = 10) underwent overnight PSG before surgical treatment. The Apnea–Hypopnea Index (AHI), Oxygen Desaturation Index (ODI), minimum and mean SpO_2_, and heart rate were recorded. Clinical features (stridor, feeding difficulties, respiratory effort) and type of surgery (supraglottoplasty [S] or supraglottoplasty with epiglottopexy [S + E]) were analyzed across LM subtypes. **Results:** Baseline AHI was significantly higher in LM Type III (25.41 ± 6.95 events/h) compared with Type II (12.50 ± 5.05) and Type I (2.84 ± 1.96; *p* < 0.001). After surgery, AHI decreased to 1.76 ± 1.56 in Type III and 0.97 ± 0.70 in Type II. ODI showed a similar trend (Type III: 9.87 ± 5.99 before vs. 0.78 ± 0.69 after surgery; *p* < 0.001). Minimum SpO_2_ increased from 69.50 ± 7.76% to 93.60 ± 1.82% in Type III (*p* < 0.001). Feeding difficulties were observed in 100% of Type III patients, compared with 83.3% of Type II and 42.9% of Type I patients. The distribution of apnea type differed significantly across groups (*p* < 0.001), with mixed obstructive–central apnea predominating in Type III. **Conclusions:** Polysomnography is an effective and objective tool for assessing LM severity and guiding surgical qualification. Increasing LM severity is associated with more pronounced PSG abnormalities, greater clinical burden, and a higher likelihood of requiring advanced surgical correction.

## 1. Introduction

Laryngomalacia (LM) is the most prevalent congenital anomaly of the upper airway and the leading cause of stridor in infancy [1,2,3]. It results from dynamic collapse of supraglottic structures during inspiration, caused by anatomical and neuromuscular immaturity [4,5]. Clinically, the condition presents with a broad spectrum of severity, ranging from benign inspiratory noise to progressive respiratory distress, hypoxemia, and feeding difficulties. While most cases resolve spontaneously, approximately 10–20% of patients develop significant morbidity requiring surgical intervention, most commonly supraglottoplasty [6,7].

Diagnosis of LM relies primarily on clinical symptoms corroborated by fiberoptic laryngoscopy or videolaryngoscopy, which demonstrate redundant supraglottic tissue and dynamic airway collapse [8]. Although these tools are considered diagnostic standards, their interpretation remains largely subjective, depending on examiner experience, the infant’s state during examination, and the absence of standardized grading systems. Moreover, endoscopy is typically performed while the child is awake and therefore cannot fully assess the functional impact of airway obstruction during sleep [9,10,11,12].

Sleep-disordered breathing (SDB), including obstructive sleep apnea (OSA), has been increasingly recognized in infants with LM, particularly in more severe cases. However, the routine use of polysomnography (PSG)—the gold standard for diagnosing SDB—remains limited in this population [13,14,15,16]. Previous studies, such as those by Manickam et al., have demonstrated the utility of PSG in quantifying disease severity, predicting surgical benefit, and distinguishing between central and obstructive apnea component [17]. Nonetheless, PSG is still underutilized in clinical practice, and standardized criteria for its application in LM evaluation or postoperative follow-up are lacking [18,19].

Furthermore, the relationship between PSG parameters, LM subtypes (I–III), clinical manifestations, and surgical outcomes remains insufficiently defined. Existing evidence suggests that infants with more severe anatomical presentations—particularly those with epiglottic prolapse (Type III)—experience more profound oxygen desaturation, higher AHI, and an increased prevalence of mixed apneas. However, these observations are limited by small sample sizes, inconsistent use of PSG, and heterogeneity in clinical phenotyping [20,21,22].

Therefore, the aim of the present study was to evaluate the role of polysomnography as an objective tool for stratifying disease severity in infants with laryngomalacia, and to correlate PSG findings with LM subtype, clinical presentation, and type of surgical intervention. By integrating anatomical classification with functional sleep metrics, this study seeks to establish a more comprehensive framework for the evaluation and management of children with LM.

## 2. Materials and Methods

### 2.1. Study Design and Participants

This observational study included 42 infants diagnosed with congenital laryngomalacia who were evaluated and treated between 2020 and 2025 at the Department of Pediatric Otolaryngology, Institute of Pediatrics, Poznań University of Medical Sciences. The study focused on clinical features, polysomnographic findings, and surgical outcomes. Patients were stratified into three groups according to the anatomical classification of laryngomalacia determined via flexible videolaryngoscopy: type I (*n* = 14), type II (*n* = 18), and type III (*n* = 10).

Eligible participants were infants under 12 months of age with a confirmed diagnosis of laryngomalacia and complete preoperative clinical and polysomnographic documentation. Inclusion criteria required the presence of key clinical symptoms such as inspiratory stridor, increased respiratory effort, feeding difficulties, or suspicion of obstructive sleep apnea, supported by endoscopic findings. Only patients who underwent surgical treatment—supraglottoplasty alone or in combination with epiglottopexy—were included. Exclusion criteria comprised other congenital airway anomalies, syndromic diagnoses, neuromuscular disorders, craniofacial malformations, or incomplete polysomnographic data.

The study population consisted of 21 boys and 21 girls, with a mean age of 5.79 ± 2.23 months. BMI values varied according to laryngomalacia subtype, with mean values of 16.34 ± 3.84 kg/m^2^ in type I, 13.94 ± 1.97 kg/m^2^ in type II, and 13.21 ± 3.82 kg/m^2^ in type III, reflecting increasing nutritional compromise with greater disease severity.

This research was conducted in accordance with the Declaration of Helsinki and institutional ethical standards. The study protocol, involving a non-interventional analysis of routinely collected clinical and diagnostic data, was formally reviewed by the Bioethics Committee of the Poznań University of Medical Sciences. The Committee confirmed in writing that the project did not meet the criteria for a medical experiment and therefore did not require further ethical approval. This confirmation, dated 9 June 2021, was issued by the Chair of the Bioethics Committee, Prof. Maciej Krawczyński, and is archived at the study center.

### 2.2. Clinical Assessment

A comprehensive clinical evaluation was performed in all patients upon admission. The primary symptoms prompting referral included inspiratory stridor, signs of increased respiratory effort (such as suprasternal or intercostal retractions), feeding difficulties (including prolonged feeding times, choking, or failure to thrive), and parental reports suggestive of obstructive sleep apnea (OSA), such as witnessed apneic episodes or noisy breathing during sleep. A detailed clinical history was obtained from parents or caregivers, and each patient underwent physical examination by a pediatric otolaryngologist.

Anthropometric measurements, including weight and length, were recorded for all infants and used to calculate body mass index (BMI, kg/m^2^), which served as an indirect indicator of nutritional status. Notably, BMI values tended to decline with increasing anatomical severity of laryngomalacia.

All patients underwent flexible fiberoptic laryngoscopy during spontaneous breathing to confirm the diagnosis of congenital laryngomalacia. The endoscopic evaluation included dynamic assessment of supraglottic structures and was used to classify the anatomical subtype of LM. The decision regarding surgical intervention was made by a multidisciplinary team composed of pediatric otolaryngologists, pediatricians, pulmonologists, gastroenterologists, and anesthesiologists. Surgical eligibility was determined based on the severity of symptoms and endoscopic findings. Infants with mild, self-limiting symptoms were managed conservatively, whereas those with significant respiratory or feeding compromise were qualified for surgical treatment.

### 2.3. Polysomnography (PSG)

To obtain objective data supporting clinical decision-making, overnight polysomnography (PSG) was performed in all surgically treated patients on the night preceding surgery. Recordings were carried out in a pediatric sleep laboratory under standard environmental conditions. PSG was performed using a full-montage setup that included nasal airflow measurement via pressure transducer, and thermistor, thorax and abdomen respiratory inductance plethysmography, oxygen saturation (SpO_2_) via pulse oximetry, electroencephalography (EEG), electrooculography (EOG), chin and tibial electromyography (EMG), and electrocardiography (ECG).

The following parameters were extracted from the PSG recordings:

- Apnea–Hypopnea Index (AHI): the number of apneas and hypopneas per hour of sleep, used as the primary marker of sleep-disordered breathing severity;
- Oxygen Desaturation Index (ODI): the number of desaturation episodes (≥3% drop in SpO_2_) per hour;
- Minimum and mean SpO_2_;
- during total sleep time, reflecting nocturnal hypoxemia;
- Mean heart rate, as an indirect measure of sleep-related autonomic disturbance.

Sleep staging and respiratory event scoring were performed manually according to the pediatric guidelines of the American Academy of Sleep Medicine (AASM). Apneas were classified as obstructive, central, or mixed based on airflow and respiratory effort channels. The classification of the dominant type of apnea (central, obstructive, or mixed) was subsequently used to correlate with laryngomalacia subtype and determine surgical priority.

### 2.4. Surgical Intervention

All surgical procedures were performed under general anesthesia with spontaneous ventilation using a tubeless technique, ensuring an unobstructed endoscopic view of the supraglottic airway. The choice of surgical approach was individualized according to the anatomical phenotype of laryngomalacia and the severity of clinical compromise.

Infants with type I and type II LM—characterized by redundant arytenoid mucosa and shortened aryepiglottic folds, respectively—underwent standard supraglottoplasty (S). This procedure involved trimming and/or repositioning of redundant mucosa and incision of the aryepiglottic folds to enlarge the supraglottic airway. In contrast, patients with type III LM, defined by dynamic epiglottic prolapse and posterior collapse during inspiration, required a more extensive intervention consisting of supraglottoplasty combined with epiglottopexy (S + E). This combined technique stabilized the epiglottis by placing Vicryl sutures after electrocautery de-epithelialization of the tongue base and the lingual surface of the epiglottis, thereby preventing posterior displacement.

All procedures were performed under suspension microlaryngoscopy by two experienced pediatric otolaryngologists using cold steel instruments, microlaryngeal scissors, or a microdebrider.

### 2.5. Statistical Analysis

Data analysis was performed using StatPlus software (AnalystSoft, Brandon, FL, 33511, USA, https://www.analystsoft.com/en/products/statplusmacle/). The normality of distribution for continuous variables was assessed with the Shapiro–Wilk test. Variables following a normal distribution were reported as mean ± standard deviation (SD), whereas non-normally distributed variables were presented as medians with interquartile ranges.

Comparisons among the three LM subtypes were conducted using one-way analysis of variance (ANOVA). When significant differences were detected, Scheffé’s post hoc test was applied to identify specific group contrasts. Comparisons between two groups were performed with Student’s *t*-test for parametric data.

Categorical variables—including apnea type, presence of respiratory effort, feeding difficulties, suspicion of OSA, and type of surgery—were analyzed across study groups using tests appropriate for distributional characteristics. Due to the presence of small expected values (<5) and empty cells in several contingency tables, Fisher’s exact test (or its Freeman–Halton extension for r × c tables) was employed. In cases where a full Fisher’s exact test could not be implemented because of table dimensions, the likelihood-ratio chi-square test (log-likelihood) was used as a valid approximation.

A two-sided *p*-value < 0.05 was considered statistically significant.

## 3. Results

### Clinical Characteristics and Apnea Classification

Significant differences in clinical presentation and management were observed across the laryngomalacia severity groups (LM 1–3), as summarized in Table 1. Infants with mild disease (LM 1) most commonly exhibited no significant apnea or central apnea, preserved respiratory effort, and fewer feeding difficulties or suspicion of obstructive sleep apnea (OSA). By contrast, moderate (LM 2) and severe (LM 3) cases were increasingly associated with obstructive or mixed apnea, loss of respiratory effort, and a higher prevalence of feeding difficulties and suspected OSA. Although stridor was universally present across all groups, the surgical approach varied significantly: standard supraglottoplasty was performed in all LM 1 and most LM 2 patients, whereas the majority of LM 3 patients (90%) required an extended procedure involving epiglottopexy with adjunct techniques. Statistically significant associations were identified for type of apnea (*p* < 0.001), respiratory effort (*p* < 0.001), feeding difficulties (*p* < 0.01), OSA suspicion (*p* < 0.05), and type of surgery (*p* < 0.001), highlighting the progressive clinical complexity and therapeutic demands associated with increasing severity of laryngomalacia.

**Figure 1:**
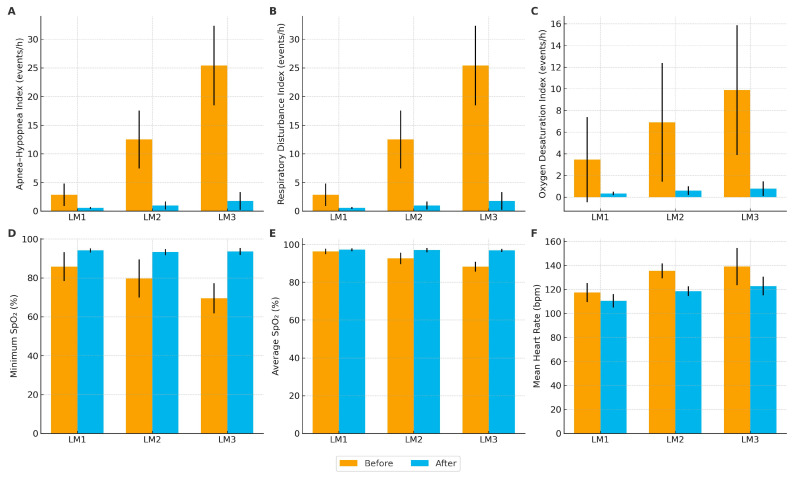
Comparison of polysomnographic and cardiopulmonary parameters before and after surgical treatment across laryngomalacia subtypes (LM1–3). Panels illustrate mean values ± SD of (**A**) Apnea–Hypopnea Index (AHI), (**B**) Respiratory Disturbance Index (RDI), (**C**) Oxygen Desaturation Index (ODI), (**D**) minimum oxygen saturation (SpO_2_ min), (**E**) average oxygen saturation (SpO_2_ avg), and (**F**) mean heart rate (HR) in infants with laryngomalacia stratified by subtype (LM1–3). Blue bars represent preoperative (“Before”) values, and orange bars postoperative (“After”) values.

As shown in Figure 1, significant improvements were observed in key polysomnographic and cardiopulmonary parameters following surgical treatment across all laryngomalacia severity groups (LM 1–3), with distinct baseline differences reflecting disease severity. Prior to surgery, patients with LM 3 exhibited markedly higher AHI, RDI, and ODI values compared with LM 1 and LM 2 (all *p* < 0.001). Minimal and mean SpO_2_ values were significantly lower in LM 3, indicating more severe hypoxemia (*p* < 0.01 for LM 1 vs. LM 3 and LM 2 vs. LM 3), while mean heart rate was elevated in both LM 3 and LM 2 compared with LM 1 (*p* < 0.05). Postoperatively, a substantial reduction in AHI, RDI, and ODI was observed in all groups, accompanied by normalization of mean SpO_2_ (>96%) and improvement in heart rate. Importantly, intergroup differences diminished after surgery, underscoring the therapeutic effectiveness of both standard and extended interventions. Detailed numerical data with 95% confidence intervals are provided in Table A1.

In turn, as shown in Table A2, statistically significant improvements were observed in all evaluated sleep-related and cardiopulmonary parameters across LM 1–3 following surgical treatment. The AHI and RDI decreased significantly in all groups (*p* ≤ 0.001), reflecting a marked reduction in sleep-disordered breathing events. The ODI also improved, with the most pronounced effect in the most severe group (LM 3; *p* < 0.001). Minimum SpO_2_ levels increased significantly after surgery in all groups (*p* < 0.001), indicating improved nocturnal oxygenation. Mean SpO_2_ likewise rose significantly, particularly in LM 2 and LM 3 (*p* < 0.001), with a modest but still significant increase in LM 1 (*p* < 0.05). Mean heart rate during sleep declined significantly across all groups postoperatively, with the greatest reductions observed in LM 1 and LM 2 (*p* < 0.001), and a notable decrease also evident in LM 3 (*p* < 0.01).

## 4. Discussion

This study provides important evidence on the PSG characteristics of infants with congenital LM, stratified by anatomical subtype, and their correlation with clinical features and surgical outcomes. Our data not only confirm the high prevalence of SDB, particularly OSA, in this population, but also provide detailed insight into how different anatomical variants influence the severity of respiratory compromise and necessitate tailored therapeutic strategies.

Our findings align closely with and expand upon those of Cortes et al., who demonstrated significant reductions in AHI and hypopnea indices after supraglottoplasty in a small cohort of nine infants [17]. By stratifying patients according to LM subtype and including a larger cohort (*n* = 42), our study builds on these data, offering a more nuanced perspective on how the anatomical pattern of supraglottic collapse—particularly epiglottic prolapse (Type III)—correlates with PSG severity. This is consistent with recent observations by Rachmawati et al., who highlighted that distinct epiglottic collapse patterns strongly determine clinical severity in sleep-disordered breathing [23]. Together, these findings support pathophysiological models emphasizing the central role of dynamic upper airway obstruction during sleep in LM-related OSA [24,25].

Furthermore, our demonstration that Type III LM is associated not only with higher baseline AHI and oxygen desaturation but also with mixed apnea patterns corroborates the neurological theory of LM proposed by Blekic et al., which suggests immaturity or dysregulation of central airway control pathways as a contributing mechanism [26]. These findings mirror the frequent coexistence of central apneas reported by Thanphaichitr et al. [27] and Verkest et al. [28], while also echoing the longitudinal variability of PSG indices reported by Um et al. [22]. Collectively, these studies suggest that mixed apneic phenotypes may serve as a clinical marker of unstable neuro-respiratory regulation in LM.

The observed improvements in AHI, ODI, and minimum SpO_2_ following surgical correction, particularly in patients who underwent combined supraglottoplasty and epiglottopexy, highlight the effectiveness of tailored surgical interventions in mitigating SDB burden [29,30,31]. Our results underscore that in Type III LM, where epiglottic prolapse contributes to posterior glottic obstruction, isolated supraglottoplasty may be insufficient. This aligns with recent surgical literature emphasizing the importance of individualized strategies, including epiglottopexy, for managing severe cases, complementing broader advances in targeted OSA surgery [32,33].

The improvements in sleep efficiency observed postoperatively are particularly noteworthy [34]. Beyond their mechanical implications, these suggest a restoration of normal sleep architecture—a finding with important developmental consequences. Recent studies show that disrupted sleep in early childhood is associated with long-term cognitive, behavioral, and emotional difficulties [35,36]. Thus, early correction of LM-associated SDB may not only improve respiratory symptoms but also support healthy neurodevelopmental trajectories [36].

From a broader clinical and health system perspective, these findings highlight an urgent need to integrate PSG into routine LM assessment protocols. Currently, many centers rely solely on endoscopic visualization to determine LM severity. While useful, this approach underestimates the functional impact of LM during sleep and may delay timely intervention [28,37,38,39]. As our data demonstrate, infants with similar endoscopic features may exhibit markedly different apnea profiles, emphasizing the necessity of a multimodal assessment strategy.

Moreover, our findings have implications for feeding assessment and nutritional surveillance. The inverse relationship between LM severity and BMI in our cohort supports previous studies linking airway obstruction with increased caloric expenditure and feeding inefficiency [40,41]. Nutritional compromise in early infancy carries long-term risks for stunting and metabolic dysfunction, making early identification and correction of LM essential not only for airway health but also for overall systemic development [42,43].

The observed shift in apnea pattern (from obstructive–central to predominantly obstructive) after surgery, especially in Type III LM, supports the hypothesis that mechanical obstruction exacerbates or unmasks central instability in respiratory control [44]. This may be conceptualized through the “loop gain” framework, where increased upper airway resistance contributes to unstable feedback loops in respiratory control systems. Postoperative reduction in airway resistance likely stabilizes these loops, improving both ventilation and sleep structure [45,46,47].

Additionally, the frequent occurrence of mixed apneas in more severe LM phenotypes may indicate a functional overlap between neurogenic and anatomic etiologies—supporting an integrated “neuromechanical” model of LM pathophysiology that warrants further investigation [48,49,50]. This model aligns with emerging evidence from transcriptomic and neuroanatomical studies implicating delayed laryngeal innervation and aberrant reflex pathways in LM pathogenesis [51,52].

While this study is strengthened by its sample size, LM subtype stratification, and the use of full-montage PSG, several limitations warrant acknowledgment. First, the follow-up period was limited to the early postoperative window. Although this design allowed us to evaluate short-term surgical outcomes with precision, it does not provide information on the long-term stability of PSG improvements, growth trajectories, or neurodevelopmental outcomes. Future prospective studies with serial PSG assessments and developmental follow-up are needed to determine whether the benefits observed are sustained.

Second, our study cohort included only surgically treated infants with moderate-to-severe LM. This was an intentional design choice, as the primary objective was to quantify the effects of surgical intervention on PSG and clinical outcomes in the highest-risk group. Excluding conservatively managed patients ensured methodological homogeneity and allowed us to demonstrate clear pre- and postoperative changes. Nevertheless, this selection introduces a degree of bias and limits the generalizability of our findings to the broader LM spectrum, particularly to milder cases, which are typically managed conservatively and rarely require PSG-based decision-making.

Third, the study was conducted at a single tertiary care center, which may reduce external validity and calls for multicenter collaborations to confirm the reproducibility of our results. Finally, we did not incorporate advanced endotyping methods, such as neuroimaging, cytokine profiling, or transcriptomic analysis, which could have provided additional mechanistic insight.

Several avenues for future research emerge from this work. Prospective longitudinal follow-up with repeated PSG and standardized growth and neurodevelopmental assessments would help clarify the sustained benefits of early surgical intervention. In addition, the development of a composite LM severity score that integrates anatomical subtype, PSG indices, and clinical features represents a promising step toward personalized treatment. While our dataset offers preliminary support for this approach, validation will require larger, multicenter cohorts that also include non-surgical patients. Advances such as transcriptomic profiling of the airway mucosa, dynamic MRI, or machine learning–based endoscopy analysis could further refine disease classification and surgical planning. Finally, health-economic studies evaluating the developmental and systemic return of routine PSG in LM workup could inform future clinical guidelines and reimbursement policies.

## 5. Conclusions

This study confirms that polysomnography (PSG) is an indispensable tool in the evaluation of infants with congenital laryngomalacia. PSG provides objective quantification of sleep-disordered breathing severity and reveals clinically meaningful differences across LM subtypes, with the most pronounced abnormalities observed in Type III cases [ref]. Importantly, the postoperative improvements in PSG across all severity groups affirm its value not only in preoperative assessment but also in monitoring surgical outcomes. Given its capacity to guide clinical decision-making, predict surgical needs, and assess functional impairment during sleep, PSG should be regarded as a standard component of the diagnostic workup for infants with suspected moderate-to-severe laryngomalacia Early integration of PSG into routine evaluation enables more accurate disease stratification, facilitates timely surgical intervention, and ultimately improves patient outcomes

## Figures and Tables

**Table 1:** Comparison of Clinical Characteristics and Treatment Approaches Across Laryngomalacia Severity Groups.

		LM 1 (*n* =14)	LM 2 (*n* =18)	LM 3 (*n* =10)	*p*-Value
Type of Apnea	Central	3 (21.4%)	0 (0%)	1 (10%)	<0.001
	Obstructive	1 (7.10%)	9 (50%)	3 (30%)	
	Obstructive and Central	1 (7.10%)	8 (44.40%)	6 (60%)	
	No Significant Apnea	9 (64.30%)	1 (5.60%)	0 (0%)	
Stridor	Present	14 (100%)	18 (100%)	10 (100%)	-
	Not present	0 (0%)	0 (0%)	0 (0%)	
Respiratory effort	Present	11 (78.60%)	1 (5.60%)	0 (0%)	<0.001
	Not present	3 (21.40%)	17 (94.40%)	10 (100%)	
Feeding difficulties	Present	6 (42.90%)	15 (83.30%)	10 (100%)	<0.01
	Not present	8 (57.10%)	3 (16.70%)	0 (100%)	
OSA suspicion	Present	8 (57.10%)	14 (77.80%)	10 (100%)	<0.05
	Not present	6 (42.905)	4 (22.20%)	0 (0%)	
Type of Surgery	Standard Surgery/Supraglottoplasty	14 (100%)	17 (94.40%)	1 (10%)	<0.001
	Surgery + Epiglottopexy	0 (0%)	1 (5.60%)	9 (90%)	

Values are expressed as number and percentage within each group: *n* (%). *p*-values were calculated using Fisher’s exact test or chi-square test with likelihood ratio approximation, depending on cell count conditions. LM 1–3, Laryngomalacia severity classification groups; OSA, Obstructive Sleep Apnea.

## Data Availability

The data presented in this study are available in this article.

## Abbreviations

The following abbreviations are used in this manuscript:

AHI	Apnea–Hypopnea Index
RDI	Respiratory Disturbance Index
ODI	Oxygen Desaturation Index
SpO_2_	Peripheral capillary oxygen saturation
HR	Heart Rate
LM	Laryngomalacia
OSA	Obstructive Sleep Apnea
PSG	Polysomnography
SD	Standard Deviation
CI	Confidence Interval
BMI	Body Mass Index
SDB	Sleep-Disordered Breathing
CPAP	Continuous Positive Airway Pressure
REM	Rapid Eye Movement
NREM	Non-Rapid Eye Movement
IQR	Interquartile Range

## Appendix A

**Table A1:** Comparison of polysomnographic and cardiopulmonary parameters before and after surgical treatment in laryngomalacia patients across severity groups (LM 1–3).

Parameter			LM1	LM2	LM3	Significant Differences
**AHI**	before	Mean ± st. dev	2.84 ± 1.96	12.50 ± 5.05	25.41 ± 6.95	1 vs. 2; 1 vs. 3; 2 vs. 3
		95%Cl	1.71–3.97	9.99–15.10	20.44–30.78	
	after	Mean ± st. dev	0.54 ± 0.18	0.97 ± 0.70	1.76 ± 1.56	1 vs. 2; 1 vs. 3; 2 vs. 3
		95%Cl	0.10–0.90	0.31–1.62	0–3.79	
**RDI**	before	Mean ± st. dev	2.84 ± 1.96	12.50 ± 5.05	25.41 ± 6.95	1 vs. 2; 1 vs. 3; 2 vs. 3
		95%Cl	1.71–3.97	9.99–15.10	20.44–30.78	
	after	Mean ± st. dev	0.54 ± 0.18	0.97 ± 0.70	1.76 ± 1.56	1 vs. 2; 1 vs. 3; 2 vs. 3
		95%Cl	0.10–0.90	0.31–1.62	0–3.79	
**ODI**	before	Mean ± st. dev	3.46 ± 3.93	6.90 ± 5.49	9.87 ± 5.99	1 vs. 3
		95%Cl	1.20–5.74	4.17–9.63	5.58–14.16	
	after	Mean ± st. dev	0.34 ± 0.17	0.60 ± 0.41	0.78 ± 0.69	1 vs. 3
		95%Cl	0–0.75	0.22–0.98	0–1.62	
**SpO_2_ min (%)**	before	Mean ± st. dev	85.79 ± 7.44	79.72 ± 9.82	69.50 ± 7.76	1 vs. 3; 2 vs. 3
		95%Cl	81.49–90.08	74.83–84.61	63.94–75.06	
	after	Mean ± st. dev	94.12 ± 1.09	93.29 ± 1.61	93.60 ± 1.82	-
		95%Cl	92.04–95.96	91.80–94.77	91.34–98.86	
**Sp0_2_ average**	Before	Mean ± st. dev	96.29 ± 1.44	92.61 ± 3.03	88.20 ± 2.57	1 vs. 2; 1 vs. 3; 2 vs. 3
		95%Cl	95.46–97.12	91.11–94.12	86.36–9004	
	after	Mean ± st. dev	97.19 ± 0.81	97.00 ± 1.16	96.80 ± 0.84	-
		95%Cl	95.44–98.56	95.93–98.07	95.77–97.84	
**Mean heart rate**	before	Mean ± st. dev	117.35 ± 7.91	135.44 ± 6.22	139.10 ± 15.51	1 vs. 2; 1 vs. 3
		95%Cl	112.79–121.92	132.35–138.53	128.00–150.19	
	after	Mean ± st. dev	110.50 ± 5.60	118.57 ± 4.12	122.80 ± 7.82	1 vs. 2; 1 vs. 3
		95%Cl	100.20–119.80	114.77–122.38	113.09–132.51	

AHI, Apnea–Hypopnea Index; RDI, Respiratory Disturbance Index; ODI, Oxygen Desaturation Index.

## Appendix B

**Table A2:** Pre- vs. Postoperative Changes in Sleep-Related and Cardiopulmonary Parameters Across Laryngomalacia Severity Groups (LM 1–3).

Parameter	LM	Comparison	t-Student *p*-Value
AHI	1	After vs. before	<0.001
	2	After vs. before	0.001
	3	After vs. before	0.001
RDI	1	After vs. before	0.001
	2	After vs. before	0.001
	3	After vs. before	0.001
ODI	1	After vs. before	<0.05
	2	After vs. before	<0.01
	3	After vs. before	<0.001
SpO_2_ min (%)	1	After vs. before	<0.001
	2	After vs. before	<0.001
	3	After vs. before	<0.001
SpO_2_ average	1	After vs. before	<0.05
	2	After vs. before	<0.001
	3	After vs. before	<0.001
Heart Rate (bpm)	1	After vs. before	<0.001
	2	After vs. before	<0.001
	3	After vs. before	<0.01

AHI, Apnea–Hypopnea Index; RDI, Respiratory Disturbance Index; ODI, Oxygen Desaturation Index.
